# Pedicled gastric seromuscular patch for one-stage closure of a bronchopleural fistula: a case report

**DOI:** 10.1186/s40792-018-0444-1

**Published:** 2018-04-20

**Authors:** Jun Suzuki, Hiroyuki Oizumi, Hirohisa Kato, Akira Hamada, Hikaru Watarai, Kenta Nakahashi, Takayuki Sasage, Mitsuaki Sadahiro

**Affiliations:** Second Department of Surgery, Faculty of Medicine, Yamagata University, 2-2-2 Iida-Nishi, Yamagata, 990-9585 Japan

**Keywords:** Bronchopleural fistula, One-stage closure, Gastric seromuscular patch, Omental pedicle flap

## Abstract

**Background:**

One-stage closure and fenestration are the available surgical options for bronchopleural fistula (BPF). One-stage closure may be applicable in cases with favorable infection control. Closing the bronchopleural stump is difficult due to thick adhesion caused by inflammation and a high risk of pulmonary artery injury. We report the successful closure of a BPF using a gastric seromuscular patch with an omental pedicle flap.

**Case presentation:**

A 73-year-old man underwent right lower lobectomy with ND2a-2 lymph node dissection for lung adenocarcinoma. He was admitted to a local hospital for pneumonia. Three days after admission, his thoracic cavity was drained and a BPF was suspected. During the primary operation, the latissimus dorsi muscle and anterior serratus muscle were dissected via posterolateral incision, and we decided to close the fistula using the gastric seromuscular layer and omental pedicle flap. The patient was discharged 20 days after surgery. After 2 years, he has not had cancer recurrence and currently leads an active life.

**Conclusions:**

This method provided immediate airtight closure and luminal opening of the middle bronchus in our patient with a large BPF and appeared superior to using the omentum alone. This procedure is useful for one-stage closure and does not require fenestration in cases with favorable infection control.

## Background

Bronchopleural fistulas (BPF) after lung surgery are difficult to treat and have a high mortality rate [1]. One-stage closure may be applicable in cases with favorable infection control. Closing the bronchopleural stump is difficult because of thick adhesions caused by inflammation, and there is a high risk of damaging the pulmonary artery near the stump. We report a case of BPF closure using combined gastric seromuscular patch with omental pedicle flap.

## Case presentation

A 73-year-old man exhibited an abnormal shadow on plain chest radiography during a medical examination. Chest computed tomography (CT) revealed a 27-mm solid mass shadow in the right lower lobe, and lung adenocarcinoma was detected on bronchoscopy and was diagnosed as cT1cN0M0. Preoperative forced vital capacity and forced expiratory volume in 1 s (FEV1.0) were 2390 and 1550 mL, respectively. His medical history was remarkable for hypertension, with no history of diabetes and steroid use.

After consulting with the patient and his family, he selected an open surgery. Therefore, posterolateral thoracotomy was performed through the fifth intercostal space, and right lower lobectomy and mediastinal lymph node dissection (ND2a-2) were performed. We dissected the bronchus using a linear stapler, and the surgery was completed without complications. Intraoperative volume of blood loss was 198 mL, and the volume of infusion was 2002 mL. Oral intake restarted the next morning. The chest drain was removed on postoperative day 1, and the patient was discharged on postoperative day 7. The final pathological stage was pT1bN2M0, pStage IIIA.

**Fig. 1:**
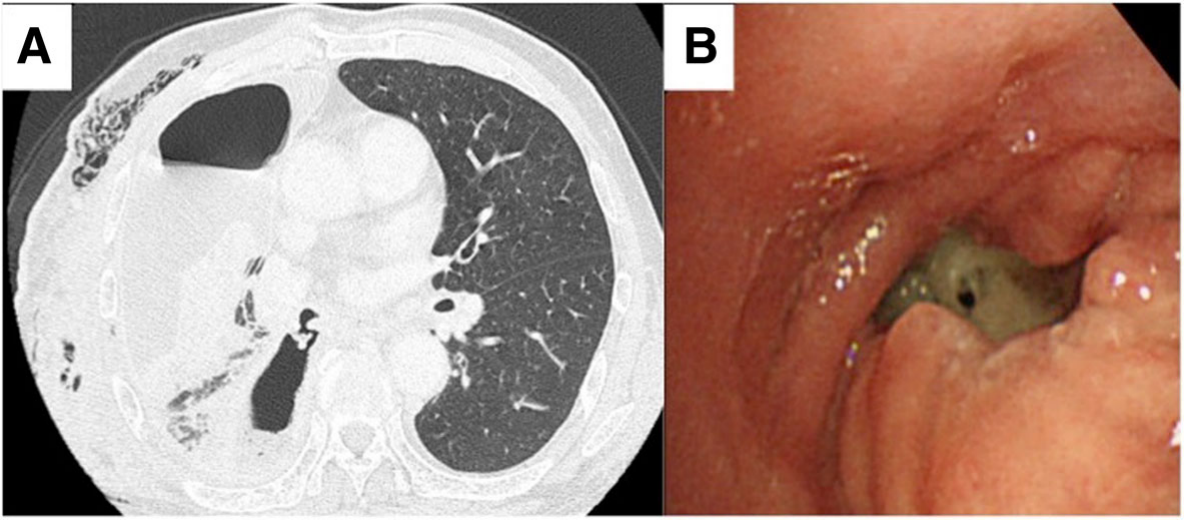
Detection of the bronchopleural fistula (BPF) via bronchoscopy and computed tomography (CT). **a** Chest CT showed the BPF but no aspiration pneumonia. **b** Bronchoscopy showed the BPF at the right lower bronchial stump

Six days after discharge, he was admitted to a local hospital for pneumonia. Three days after admission, his thoracic cavity was drained and a BPF was suspected. The patient was transferred to our hospital, and chest CT revealed a right BPF (Fig. 1a). The laboratory findings on admission were as follows: hemoglobin, 12.0 g/dL; white blood cell count, 10,900/mm^3^ with 91.6% neutrophils and 6.0% lymphocytes; platelet count, 544,000/mm^3^; total protein, 5.4 g/dL; albumin, 2.3 g/dL; and blood sugar, 145 mg/dL. Bronchoscopy showed that the BPF was located at the bronchial stump (Fig. 1b). Therefore, we planned an emergent one-stage surgery because the patient did not have aspiration pneumonia or systemic inflammatory response syndrome.

**Fig. 2:**
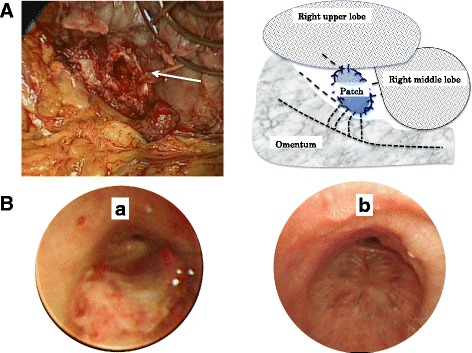
Intraoperative and postoperative findings. **A** Intraoperative photograph and schema showed a BPF (arrow) in the right lower bronchus. The schema of the bronchial stump was closed using a pedicled gastric seromuscular flap and the omentum. **B** Bronchoscopy showed the patch closure using the gastric seromuscular layer. *a* 5 days after surgery. *b* 125 days after surgery

The patient was placed in the left hemi-lateral position, and thoracotomy was performed through the fifth intercostal space, similar to the initial operation. There were many strong adhesions in the thoracic space. The site of the BPF was identified after debridement of necrotic tissue, which was 7 × 14 mm in diameter (Fig. 2a). We initially planned to resect at the truncus intermedius and perform a right middle lobectomy; however, this was impossible due to the strong adhesions. Therefore, during the primary operation, the latissimus dorsi muscle and anterior serratus muscle were dissected via posterolateral incision, and we closed the fistula using the gastric seromuscular layer and omental pedicle flap.

The abdomen was opened via vertical midline incision, and the omentum was separated from the attachments to the transverse colon and stomach. We ligated the left gastroepiploic artery and created a pedicled gastric seromuscular patch that was 20 × 20 mm in size. The defect in the gastric wall was sutured, and the flap was transferred trans-diaphragmatically into the chest. The gastric artery rami of the left gastroepiploic artery supplied blood to the patch, which was kept in contact with the right gastroepiploic artery and omental vessels. The patch was sutured to the BPF using running 4-0 monofilament sutures, and the omentum was placed inside the residual thoracic cavity (Fig. 2a). The operation lasted 277 min, intraoperative blood loss was 474 g, and postoperative vital signs were stable.

To prevent pressure around the stump closure, differential lung ventilation was performed for 7 days. H2 blocker was administered to prevent gastric ulcer or gastric rupture.

Postoperative bronchoscopy showed that the BPF was successfully closed (Fig. 2b); however, *Corynebacterium* was detected on tissue specimen that was obtained during surgery. In response, carbapenem antibiotics were administered for 10 days following the operation, and inflammation was eventually controlled. The patient was discharged 20 days after surgery. After 2 years, he has not had cancer recurrence and currently leads an active life.

## Discussion

BPFs that appear after lung surgery are difficult to treat and have a high mortality rate [1]. Appropriate drainage is required to prevent aspiration pneumonia. Additionally, surgeons need to determine whether to perform a one-stage closure or fenestration to treat the BPF. Small fistulas can be treated via transbronchial approach [2, 3], whereas large fistulas or fistulas in cases with concurrent pyothorax require fenestration. One-stage closure may be applicable in cases with favorable infection control.

In this report, our case was not complicated by aspiration pneumonia or systematic inflammatory response syndrome, and re-expansion via drainage was successful. Base on these findings, we performed a one-stage closure of the BPF. Closure was expected to be difficult for three reasons: (1) the pulmonary artery and bronchopleural stump were contiguous, (2) the fistula was too large to close easily, and (3) the middle lobe bronchus was near the BPF. We initially considered performing a middle lobectomy; however, we rejected this option due to the presence of thick adhesions in the hilum. During the primary operation, the latissimus dorsi muscle and anterior serratus muscle were dissected via posterolateral incision; therefore, it was difficult to cover or wedge the bronchopleural stump using the muscle flaps.

Using pedicled omentum to treat BPF cases with concurrent pyothorax results in a favorable outcome because this method provides excellent infection control and improves local blood flow. However, if the fistula is large, it is difficult to obtain an airtight closure. A flap composed of the greater omentum and a segment of the greater curvature of the right gastroepiploic artery was first described in 1977 [4]. In previous studies, the maximum length of the pedicle was 30 cm when the omental arcade was used, and in plastic surgery, the maximum size of gastric patches was approximately 10 × 10 cm [5, 6]. The use of pedicled omentum in cases of BPF with concurrent chronic pyothorax, or in those requiring a second closure after fenestration, has been reported. In our case, the size of the BPF was 7 × 14 mm, and the 20 × 20-mm gastric seromuscular patch provided immediate airtight closure of the BPF. Therefore, our method appears superior to those using the omentum alone in patients with little omental tissue. To our knowledge, this is the first report to determine that a pedicled gastric seromuscular flap combined with the omentum was useful for one-stage closure of a BPF.

## Conclusions

In summary, pedicled gastric seromuscular patch provided immediate airtight closure in a patient with a large BPF and appeared superior to using the omentum alone. This procedure is useful for one-stage closure and does not require fenestration in cases with favorable infection control.

## Abbreviations

BPF: Bronchopleural fistula
CT: Computed tomography
